# Characterization and genomic analysis of DSF2: a novel lytic phage infecting multidrug-resistant *Shigella*

**DOI:** 10.3389/fmicb.2026.1740847

**Published:** 2026-04-24

**Authors:** Shaofu Du, Huiqun Jia, Huanhuan Lu, Xiaoying Li, Mingjuan Yang, Hui Zhang, Hao Wu, Ligui Wang, Binghua Zhu

**Affiliations:** 1 School of Public Health, Southern Medical University, Guangzhou, China; 2 Department of Infectious Disease Prevention and Control, Chinese PLA Center for Disease Control and Prevention, Beijing, China; 3 School of Public Health, Chinese Medical University, Shenyang, China; 4 School of Public Health, Zhengzhou University, Zhengzhou, China; 5 Department of Disease Prevention and Control, The 305 Hospital of PLA, Beijing, China

**Keywords:** comparative genomics, genome sequencing, headmorphology, lytic phage, *Shigella flexneri*

## Abstract

**Background:**

Multidrug-resistant *Shigella flexneri* (MDR *S. flexneri*) serotype 2a is the predominant cause of shigellosis in China, presenting a major public health challenge amid escalating antibiotic resistance and limited treatment options. Bacteriophages are gradually emerging as a highly promising alternative to antibiotics because of its highly specific bactericidal ability. However, only 113 *Shigella* phage genomes are available in NCBI as of August 2025, highlighting the need for novel lytic phages targeting prevalent MDR strains.

**Methods:**

A novel lytic phage, vB_SflP_DSF2 (DSF2), was isolated from untreated sewage at the 305 Hospital of PLA using MDR *S. flexneri* 2a strain 301 as host. Morphology was examined by transmission electron microscopy. Host range and efficiency of plating were determined against 41 bacterial strains (33 *Shigella*, 6 *Escherichia coli*, and others) using double-layer agar spot assays. One-step growth curves, pH and thermal stability, and biological properties were assessed using standard plaque assays. The complete genome was sequenced via Illumina NovaSeq, with comparative genomic and phylogenetic analyses performed using VIRIDIC, TerL phylogeny, AlphaFold structural predictions, and Swiss-Model for protein structure comparisons.

**Results:**

The DSF2 is a Schitoviridae phage with an elongated prolate head, short non-contractile tail. It produces haloed 1–2 mm plaques indicating depolymerase activity, with a 60-min latent period and 115 PFU/cell burst size. The DSF2 remains stable from 4 °C to 50 °C and active at pH 4–10, selectively lysing all *S. flexneri* serotype 2/X strains. Genomic analysis revealed that DSF2 possesses a 72,532 bp dsDNA genome with a G+C content of 44.89%, containing 89 predicted open reading frames. The DSF2 harbors no virulence or antibiotic resistance genes. Closest relative *Shigella* virus Moo19 shares 94.1% identity, defining the DSF2 as a new species. The prolate head of DSF2 closely resembles that of *Escherichia coli* phage PH444, driven by divergent Hoc-like head decoration, despite the conservation of capsid and portal proteins when compared to *Shigella* virus Moo19.

**Conclusion:**

The DSF2 represents a novel Schitoviridae species that expands the limited *Shigella* phage repertoire, offering precision biocontrol against MDR *S. flexneri* serotype 2/X with minimal microbiome disruption. Hoc-like head decoration likely drives DSF2's unique prolate morpholog through intercapsomer angular constraints.

## 1 Introduction

*Shigella* is among the most prevalent etiological agents of diarrhea in developing and underdeveloped countries, primarily impacting children under 5 years of age (Cohen et al., 2022). It is mainly transmitted via the fecal-oral route, and an extremely low infectious dose (10–100 bacterial cells) is sufficient to cause infection. The genus *Shigella* comprises four species—*S. flexneri, Shigella sonnei, Shigella dysenteriae*, and *Shigella boydii*—which are further divided into serotypes and sub-serotypes based on O antigen repeats of lipopolysaccharide (Hendrick et al., 2025). In China, the distribution of *Shigella* species is heterogeneous, with *S. flexneri* type 2a being the most common (Zhang et al., 2021). Historically, bacillary dysentery was treated effectively with antibiotics such as ciprofloxacin, piperacillin, azithromycin, and ceftriaxone; however, extensive antibiotic use has contributed to the emergence of MDR *Shigella* strains (Sethuvel et al., 2019; Gaufin et al., 2022). As of May 17, 2024, the World Health Organization categorized fluoroquinolone-resistant *Shigella* as a high-priority pathogen for antibiotic resistance, with very few new therapeutic options currently under development (Sati et al., 2025).

Antibiotics, particularly fluoroquinolones, are commonly used to treat *Shigella* infections, but they currently face many challenges in clinical application (Laxminarayan et al., 2013). Firstly, the development of new antibiotics is a lengthy process. In addition, antibiotics often have broad-spectrum activity, which can disrupt the natural microbiota, leading to side effects such as gastrointestinal disturbances and opportunistic infections. Furthermore, bacteria can quickly acquire resistance through mutations or horizontal gene transfer, rendering many antibiotics ineffective. In contrast, phages can be rapidly isolated and characterized from the environment. Additionally, phages are highly specific to their bacterial targets, reducing the risk of disrupting the microbiome. Moreover, phages can evolve rapidly to overcome bacterial resistance, which makes them less likely to encounter resistance compared to traditional antibiotics (Olawade et al., 2024). Meanwhile, a study highlighted that phages exhibit good safety and efficacy in bacterial decolonization (Fang et al., 2024). Phages are considered a promising future hope in the fight against antibiotic resistance (Hatfull et al., 2022), with many phages, including those targeting MDR *Shigella*, being isolated and characterized. For example, the lytic phage Sfk20 targets multiple *S. flexneri* serotypes, *S. sonnei*, and *S. dysenteriae* 1 (Mallick et al., 2021) while the novel lytic phage vB_ShiP-A7 targets MDR *S. flexneri* and *Escherichia coli* (*E. coli*) (Xu et al., 2021). Although the isolation of *Shigella* phages has been an ongoing effort, as of August 2025, only 113 genome sequences of *Shigella* phages have been deposited in the NCBI database, compared to over 1,000 *E. coli* and *Salmonella* phage sequences. Therefore, the isolation and characterization of additional *Shigella*-specific phages is essential for expanding the available phage arsenal.

The newly isolated phage is classified into the same genus as known phages when its nucleotide sequence similarity exceeds 70%, and into the same species when it exceeds 95% (Adriaenssens and Brister, 2017). In this study, a new phage DSF2 was isolated from sewage at a hospital in Beijing, China, using MDR *S. flexneri* as the host. BLASTn comparison showed that the most similar phage to DSF2 was Moo19 (Subramanian et al., 2024), with a nucleotide sequence similarity of 94.1%. Further analysis using VIRIDIC indicated an intergenomic similarity of 86.2%. This suggests that DSF2 represents a new species of *Shigella* phage, enriching the *Shigella* phage database and offering a new candidate for the clinical treatment of MDR *S. flexneri* infections. The typical short-tailed phage head, such as that of Moo19, is icosahedral in shape; however, the head of DSF2 exhibits a more elongated cylindrical morphology, similar to PH444 (Zhang et al., 2024), demonstrating the diversity and complexity of phage head shapes. The genomes of Moo19, PH444, and DSF2 were compared, and further analysis of the three-dimensional structures of proteins potentially affecting the phage head morphology suggested that the Hoc-like head decoration may play a potential role in determining the head shape.

## 2 Materials and methods

### 2.1 Bacterial strains and serotyping

The bacterial strains used in this study were detailed in Table 1. *Shigella, Salmonella enterica* serovar Typhimurium (*S*. Typhimurium), and Methicillin-Resistant *Staphylococcus aureus* (MRSA) strains were stored in our laboratory, whereas *E. coli* strains were procured from Borel (Beijing) Technology Service Co. Among the *S. flexneri* strains, the serotype 2a strain 301 (SF2a301) was confirmed as MDR, exhibiting resistance to aminoglycosides, amphenicols, penicillins, quinolones, and tetracyclines (Yang et al., 2018). This strain was used for isolating the phage. The remaining 40 strains were employed to assess the host spectrum of the isolated phage.

**Table 1:** Host spectrum of DSF2.

Bacterial species	Infectivity	EOP
*S. flexneri* 1a	–	–
*S. flexneri* 2a	+	1
*S. flexneri* 3a	–	–
*S. flexneri* 1	–	–
*S. flexneri* 1c	–	–
*S. flexneri* 2b	+	0.63
*S. flexneri* 2c	+	0.81
*S. flexneri* 4	–	–
*S. flexneri* 4a	–	–
*S. flexneri* 4c	–	–
*S. flexneri* 4s	–	–
*S. flexneri* 4y	–	–
*S. flexneri* X	+	0.44
*S. flexneri* Y	–	–
*S. flexneri* 6	–	–
*S. flexneri* Z	–	–
*S. flexneri* 5b	–	–
*Shigella dysenteriae* 1	–	–
*Shigella dysenteriae* 2	–	–
*Shigella dysenteriae* 11	–	–
*Shigella sonnei*	–	–
*Shigella boydii* 1	–	–
*Shigella boydii* 2	–	–
*Shigella boydii* 3	–	–
*Shigella boydii* 4	–	–
*Shigella boydii* 5	–	–
*Shigella boydii* 8	–	–
*Shigella boydii* 9	–	–
*Shigella boydii* 10	–	–
*Shigella boydii* 12	–	–
*Shigella boydii* 13	–	–
*Shigella boydii* 14	–	–
*Shigella boydii* 18	–	–
*S*. Typhimurium	–	–
MASR	–	–
*EIEC*	–	–
*EHEC* O157:H7	–	–
*EAEC*	–	–
*ETEC*	–	–
*EPEC*	–	–
*E. coli* K12	–	–

+, lysis; –, no lysis.

### 2.2 Isolation, purification, and enrichment of phage

The DSF2 was isolated from an untreated wastewater sample collected from the 305 Hospital of PLA in Beijing, China. The collection of wastewater samples did not involve human subjects or identifiable personal information; therefore, ethical approval was not required. The wastewater sample was collected on November 20, 2024, from a sewage well located east of the North Inpatient Building of the hospital. A 500 mL sterile sampling bag was used for collection, and the sample was stored at 4 °C immediately after collection. The sample was centrifuged at 5,000 rpm for 10 min to remove bacterial debris, and the resultant supernatant was filtered through a 0.22 μm membrane. For enrichment, 10 mL of the filtrate, 10 mL of Luria-Bertani (LB) broth, and 200 μL of early-log-phase SF2a301 culture were combined and incubated at 37 °C and 180 rpm for 12 h. Following incubation, the culture was centrifuged again at 5,000 rpm for 10 min and filtered with a 0.22 μm membrane filter to obtain a phage-enriched supernatant (Hyman, 2019).

A spot assay was initially conducted to detect the presence of *Shigella* phage by applying 5 μL of the enriched phage lysate onto lawns of SF2a301 on LB agar plates. After 12 h of incubation at 28 °C, the formation of clear zones around the spot indicated phage activity against SF2a301 (Paranos et al., 2024). For phage purification, the double-layer agar method was employed: 200 μL of log-phase SF2a301, 100 μL diluted phage solution, and 6 mL of soft agar (0.6% w/v) were overlaid on LB agar plates (1.5% w/v). Single plaques were selected for propagation, and this procedure was repeated five times to ensure plaque homogeneity (Kropinski et al., 2009). The high-titer purified phage was stored at 4 °C for further analyses.

### 2.3 Transmission electron microscopy (TEM)

A single colony of SF2a301 was cultured overnight in 20 mL LB at 37 °C and 180 rpm. Subsequently, 4 mL culture were transferred into 400 mL fresh LB, and 1 mL purified DSF2 suspension was added. The mixture was incubated at 37 °C and 180 rpm for 12 h to allow complete lysis. The lysate was centrifuged at 4 °C (4,750 rpm, 20 min) to remove bacterial debris. The supernatant was treated with DNase I and RNase A (final concentration 1 μg/mL each) for 30 min at room temperature. NaCl was added to a final concentration of approximately 1 M and incubated at 4 °C for 1 h, followed by centrifugation to clarify the lysate. PEG 8000 (10%, w/v) was added to the supernatant and dissolved completely. After overnight incubation at 4 °C, the mixture was centrifuged (4 °C, 4,750 rpm, 30 min) to pellet phage particles. The pellet was resuspended in SM buffer and subjected to repeated chloroform extraction until the aqueous phase became clear (Hu and Tong, 2021). The final preparation was filtered through a 0.22 μm membrane and concentrated by centrifugation. The purified phage pellet was resuspended in SM buffer and stored at 4 °C.

The purified phage particles (approximately 10^10^ plaque forming units (PFU)/mL) were immobilized on carbon-coated copper grids and negatively stained with 2% (w/v) phosphotungstic acid. Micrographs were captured using a transmission electron microscope (HITACHI HT7700) operated at an accelerating voltage of 80 kV.

### 2.4 Host spectrum and efficiency of plating (EOP)

The host spectrum and efficiency of plating (EOP) against 41 bacterial species, as detailed in Table 1, were assessed using the double-layer agar plate method. Briefly, 200 μL of the test bacterial strain cultured overnight in LB broth was mixed with 100 μL of diluted phage solution and 6 mL of soft agar, and subsequently poured onto an LB agar plate. The plate was incubated overnight at 28 °C, after which it was examined for the presence of visible lysis zones. EOP values were determined by calculating the ratio of PFU of the phage on the susceptible bacterial strain to that on the indicator bacterial strain (SF2a301) (Kutter, 2009). Each assay was performed in triplicate.

### 2.5 One-step growth curve

To determine the optimal multiplicity of infection (MOI), serial dilutions of DSF2 were mixed with exponentially growing SF2a301 cultures (OD_600_ = 0.5) at phage-to-host ratios of 10, 1, 0.1, 0.01, 0.001, 0.0001 and 0.00001. Mixtures were incubated for 5 h at 37 °C with shaking. Each sample was centrifuged at 5,000 rpm for 10 min and filtered through 0.22 μm membranes. Phage titers were determined using the double-layer agar method, and the ratio yielding the highest titer was selected as the optimal MOI.


$$\begin{array}{c} \text{Phage}\;\text{titer}\left(\text{PFU}/\text{mL}\right)=\text{Mean numbers}\;\text{of}\;\text{phage}\;\text{plaque}\\ \times \;\text{Dilution}\;\times \;10 \end{array}$$

One-step growth experiments were performed as previously described with minor modifications. The phage solution was mixed with log-phase SF2a301 (OD_600_ = 0.5) at the optimal MOI and allowed to adsorb for 10 min at 37 °C. The mixture was centrifuged at 5,000 rpm for 5 min and the pellet was washed twice with SM buffer to remove unadsorbed phages. The pellet was then resuspended in 20 mL LB broth and incubated at 37 °C. At 30-min intervals for up to 240 min, 200 μL samples were withdrawn, centrifuged at 4 °C and 12,000 rpm for 1 min, and 100 μL supernatant was collected to determine phage titer via the double-layer agar method (Kropinski, 2018). Growth curves were generated by plotting infection time against phage titer. Three replicate samples were set at each time point.

### 2.6 Thermal and pH stability tests

Phage stability under varying temperatures was assessed by incubating 150 μL aliquots (approximately 10^10^ PFU/mL) of the phage suspension in a water bath at 4 °C, 28 °C, 37 °C, 50 °C, 60 °C, and 70 °C. Phage titers were measured at 20-min intervals over 0 to 80 min using the double-layer agar method. To evaluate long-term stability, the phage suspension was stored at 4 °C for over a year, and the titer was retitrated using the same double-layer agar method. For pH stability, 100 μL of the 10^10^ PFU/mL phage particles were suspended in 900 μL SM buffer adjusted to pH values from 1 to 12 (using HCl or NaOH), incubated at 37 °C for 1 h, and 100 μL samples were recovered for titer determination (Islam et al., 2020). Three independent replicates were performed to ensure the reliability of the results.

### 2.7 Phage nucleic acid extraction and sequencing

Nucleic acid of phage was extracted from the purified phage suspension using the TIANamp Virus DNA/RNA Fast kit (Tiangen Biotech, (Beijing) Co., Ltd) according to the instruction from the manufacturer.

The whole genome sequencing (WGS) method was employed to construct paired-end libraries of varying insert sizes using the Illumina NovaSeq platform for next-generation sequencing (NGS). Raw sequence data were filtered using fastp (version: 0.20.0; https://github.com/OpenGene/fastp) to yield high-quality reads. *De novo* assembly was conducted using SPAdes (version: 3.12.0; http://cab.spbu.ru/files/release3.12.0/manual.html), and assembled contigs were screened for depth and similarity to viral genome sequences in the NCBI NR database (version: 20171010; https://ftp.ncbi.nih.gov/blast/db/) via BLASTn (version: 2.2.31; https://blast.ncbi.nlm.nih.gov/Blast.cgi) (Altschul et al., 1990). Final genome sequences were error-corrected with Pilon (version: 1.18; https://github.com/broadinstitute/pilon), and protein-coding genes were predicted using GeneMarkS (version: 4.32; https://exon.gatech.edu/) (Besemer et al., 2001). Non-coding RNA prediction involved comparison with the Rfam database (version: 14.4; https://rfam.org/) (Kalvari et al., 2018). All findings were integrated into a standard GBK (GenBank) file, and genome maps were generated using the online Proksee platform (https://proksee.ca/; Grant et al., 2023).

### 2.8 Genomic analysis

After obtaining the complete genome sequence of DSF2, BLASTn analysis was performed to evaluate genomic similarity with related phages. Pairwise intergenomic distances and similarities among viral genomes were subsequently calculated using the VIRIDIC tool (https://rhea.icbm.uni-oldenburg.de/viridic/; Moraru et al., 2020). The replication cycle of DSF2 was predicted using PhageAI (version: 1.0.2; https://app.phage.ai/) and PhaBOX (version: 2.0; https://phage.ee.cityu.edu.hk/phabox). Potential virulence factors were screened using the VFDB database (https://www.mgc.ac.cn/VFs/main.htm; Zhou et al., 2025), while the presence of antibiotic resistance genes was assessed using the CARD database (https://card.mcmaster.ca/; Alcock et al., 2023). Protein-coding gene sequence alignments were performed using DIAMOND (http://github.com/bbuchfink/diamond; version: 0.8.36) (Buchfink et al., 2015) with the NCBI NR database. DIAMOND blastp was used to compare encoded protein sequences, with a significance threshold of 10^−6^. The best hits were selected for putative function assignment. For phylogenetic analyses, predicted ORFs, specifically the terminase large subunit (TerL), were aligned with homologs from other related phages using NCBI BLASTp. Sequences with 100% query coverage were first selected from the BLASTp results. The top two hits ranked by percent identity (99.81% and 89.43%, respectively) were identified as representatives of two distinct clusters. All sequences within these clusters were subsequently retrieved, resulting in a total of 21 sequences included for phylogenetic analysis. Among these, two bacterial homologs were identified and excluded to focus on bacteriophage-specific evolutionary relationships. Multiple sequence alignment of the TerL amino acid sequences was performed using ClustalW implemented in MEGA 12. A phylogenetic tree was constructed using the neighbor-joining method with 1,000 bootstrap replicates to assess branch support. Comparative analyses between DSF2 and related phages were visualized with EasyFig (version: 2.2.5) (Sullivan et al., 2011). Further annotations were made using Adobe Illustrator (version: 2024). Protein amino acid sequences were aligned pairwise using the NCBI BLASTp tool. The protein three-dimensional structures were predicted using the AlphaFold (https://alphafold.com/) Protein Structure Database (Jumper et al., 2021), followed by structural comparison using Swiss-Model (https://swissmodel.expasy.org/; Waterhouse et al., 2018).

### 2.9 Statistical analysis

Statistical analyses and graphical plotting were performed using GraphPad Prism (version:8.0.1). Normality was assessed using the Shapiro–Wilk test, and homogeneity of variance was evaluated using the Brown–Forsythe test prior to parametric analysis. Data with a normal distribution are presented as mean ± standard deviation (SD). Intergroup comparisons were conducted using one-way analysis of variance, followed by Tukey's multiple comparisons test for *post hoc* pairwise comparisons when a significant overall difference was detected. Data that did not follow a normal distribution are expressed as median and interquartile range. Comparisons among groups were performed using the Kruskal–Wallis test, followed by Dunn's multiple comparisons test for pairwise comparisons when appropriate. Furthermore, *p*-values ≤ 0.05 was considered statistically significant.

## 3 Results

### 3.1 Morphological characterization of phage

**Figure 1:**
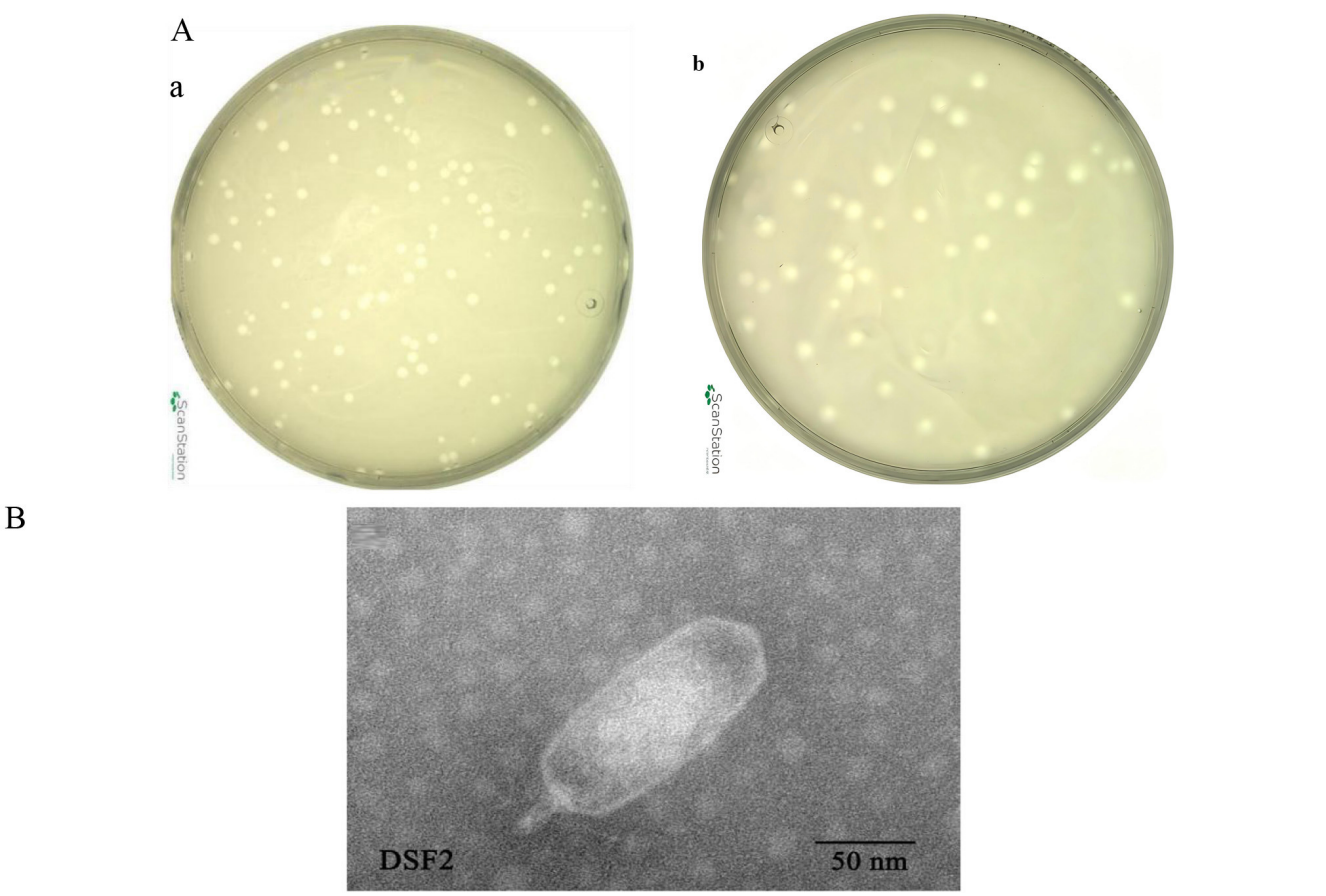
**(A)** Morphology of DSF2 phage plaques on double-layer agar at a 10–8 dilution. **(a)** Phage plaques formed by DSF2 after 12 h of incubation at 28°C, with nearly uniform size and individual plaques measuring approximately 2 mm in diameter. **(b)** Phage plaques formed by DSF2 after 24 h, with a clear halo surrounding the plaques, indicating depolymerase activity. **(B)** Electron microscopy image of DSF2 having elongated cylindrical heads and short tails. The scale bar for DSF2 is 50 nm.

A novel *Shigella*-specific phage, DSF2, was successfully isolated from hospital sewage in Beijing. DSF2 formed clear, round plaques (1–2 mm diameter) on lawns of SF2a301 after incubation at 28 °C for 12 h (Figures 1A, a). After 24 h, the plaques were surrounded by a clear halo, indicating depolymerase activity (Figures 1A, b; Latka et al., 2017). TEM revealed that the purified and concentrated DSF2 particles exhibited an elongated cylindrical capsid approximately 150 nm in diameter and a short, non-contractile tail ~20 nm in length (Figures 1B). The morphology of DSF2 closely resembles that of the PH444, which shares similar structural features, including a 100 nm capsid and a 10 nm tail. Both DSF2 and PH444 possess elongated cylindrical heads and short tails, distinct from the typical icosahedral head structure found in short-tailed phages such as Moo19. The scale bar for DSF2 and PH444 is 50 nm, while the scale bar for Moo19 is 400 Å (40 nm).

### 3.2 Host spectrum analysis

Host spectrum analysis revealed that DSF2 was capable of infecting *S. flexneri* serotypes 2a, 2b, 2c, and X (Table 1). In contrast, the *E. coli, S*. Typhimurium and MRSA were not infected. Accordingly, DSF2 exhibits a relatively narrow host spectrum and high specificity for *Shigella*. The EOP results for DSF2 across sensitive strains are summarized in Table 1.

### 3.3 One-step growth curve analysis

**Figure 2:**
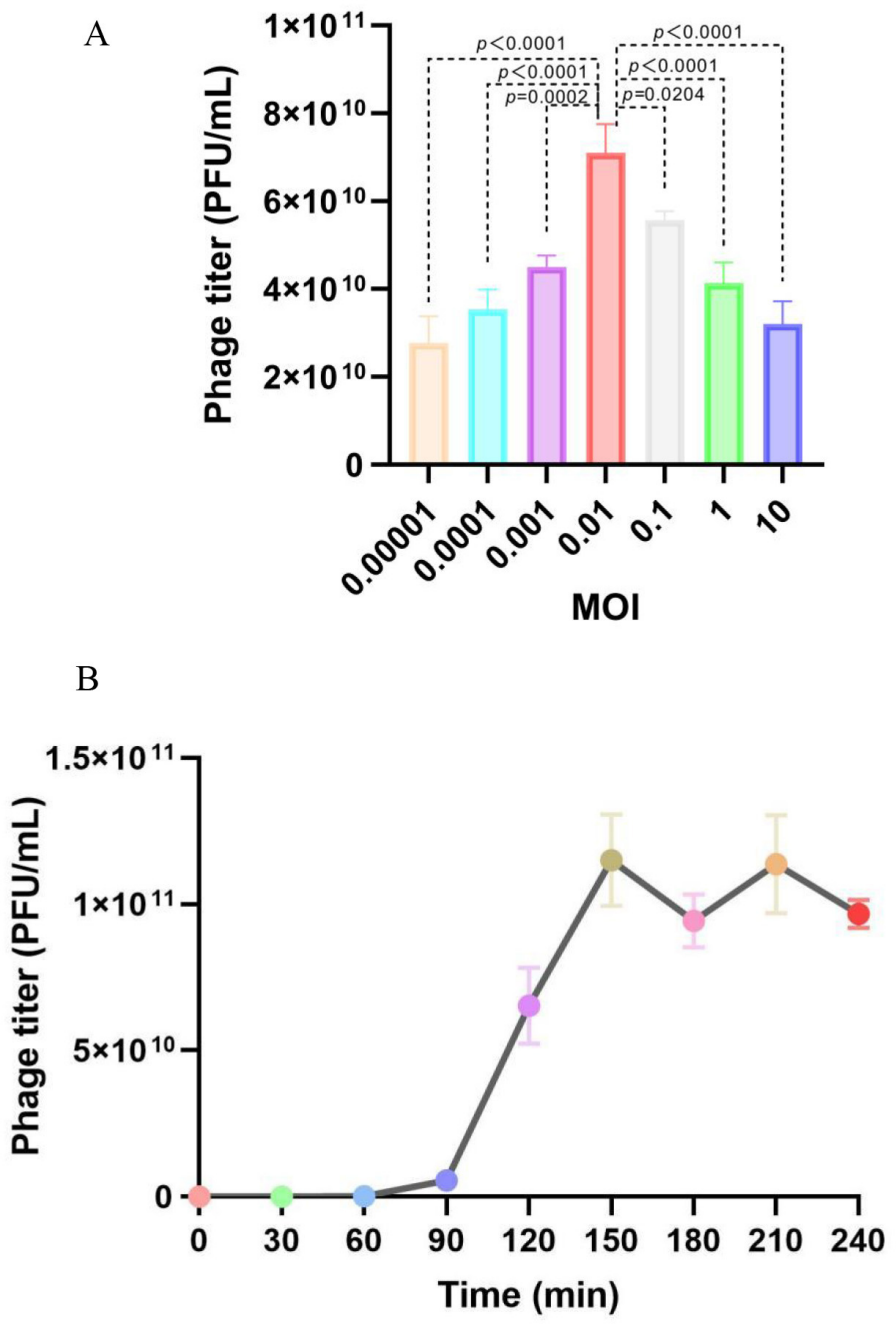
Growth characteristics of DSF2. **(A)** Assessment of optimal MOI (PFU/CFU). The optimal MOI was 0.01, yielding a phage titer of 7 × 10^10^ PFU/mL. **(B)** One-step growth curve of DSF2. SF2a301 was infected with DSF2 at an MOI of 0.01 and incubated at 37 °C. Error bars represent the SD.

MOI optimization experiments demonstrated that the highest yield was achieved at an MOI of 0.01, reaching 7 × 10^10^ PFU/mL (Figure 2A). This optimal MOI was used for subsequent one-step growth curve experiments with SF2a301 as the host. The growth curve displayed distinct lag, burst, and stationary phases. DSF2 exhibited a latent period of 60 min and a 90-min outbreak period (Figure 2B). The burst size, defined as the average number of phages produced per infected bacterial cell, was estimated by dividing the maximum phage titer (1.15 × 10^11^ PFU/mL) by the bacterial infection titer (1 × 10^9^ CFU/mL), resulting in a burst size of 115 PFU per cell.

### 3.4 Thermal and pH stability

**Figure 3:**
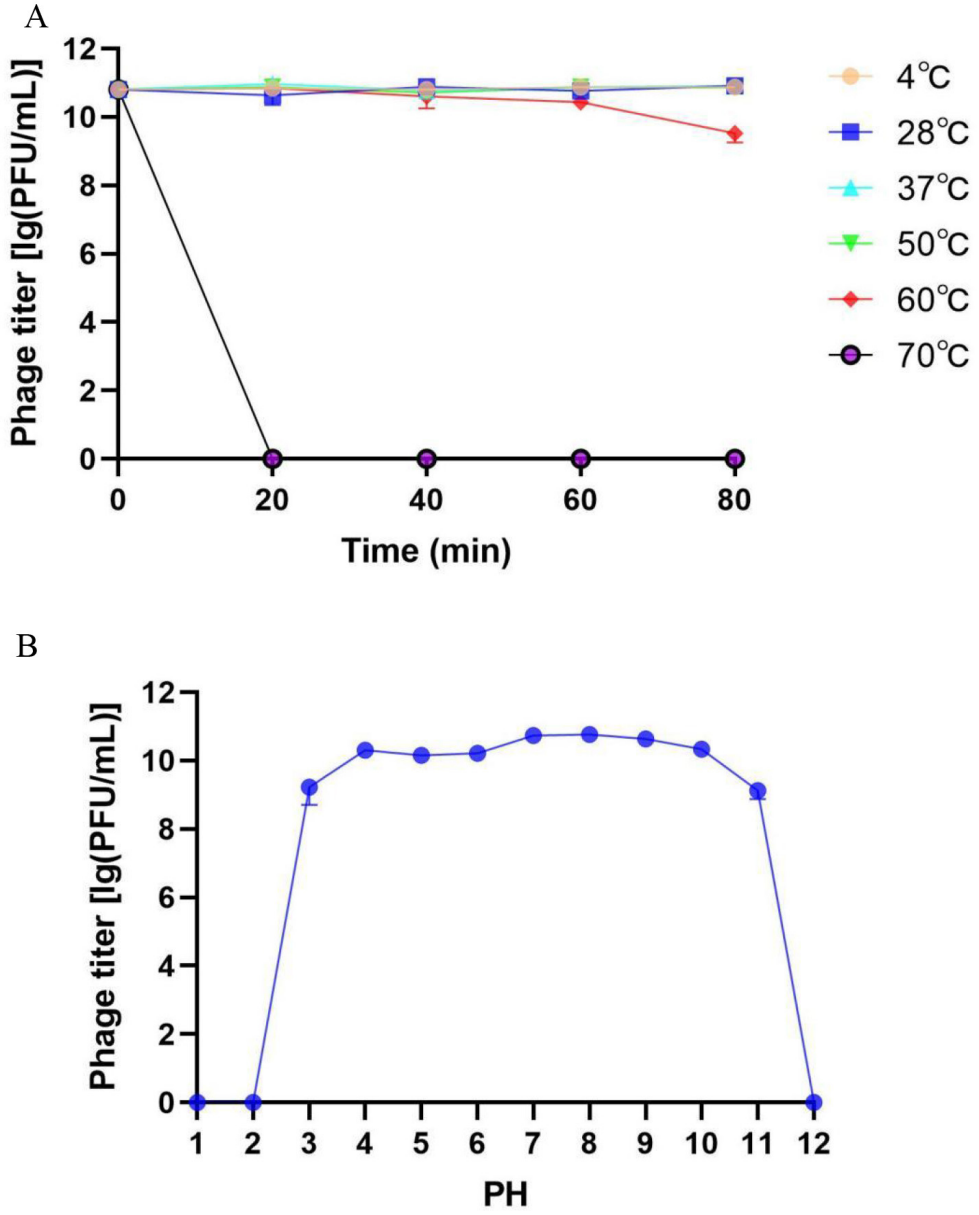
Physicochemical stability of DSF2. **(A)** Thermal stability assessment: phages incubated at various temperatures for 80 min (MOI = 0.01). **(B)** pH stability of DSF2 across a range of acidity/alkalinity (MOI = 0.01). Error bars represent the SD.

The stability of DSF2 was evaluated under different temperature and pH conditions. As shown in Figure 3A, DSF2 maintained stable within the range of 4 °C−50 °C; however, its stability began to decline after 80 min at 60 °C, with the phage titer decreasing from 2.73 × 10^10^ to 3.33 × 10^9^ PFU/mL, corresponding to an 87.8% reduction. At 70 °C, the phage stability rapidly diminished. The phage titer remained stable at approximately 5 × 10^10^ PFU/mL after 1 year of storage at 4 °C. In terms of pH stability, DSF2 maintained stable within the pH range of 4–10, while its stability diminished under increasingly acidic or alkaline conditions (Figure 3B). Complete inactivation occurred when the pH below two or exceeded 11.

### 3.5 Basic genomic features of DSF2

**Figure 4:**
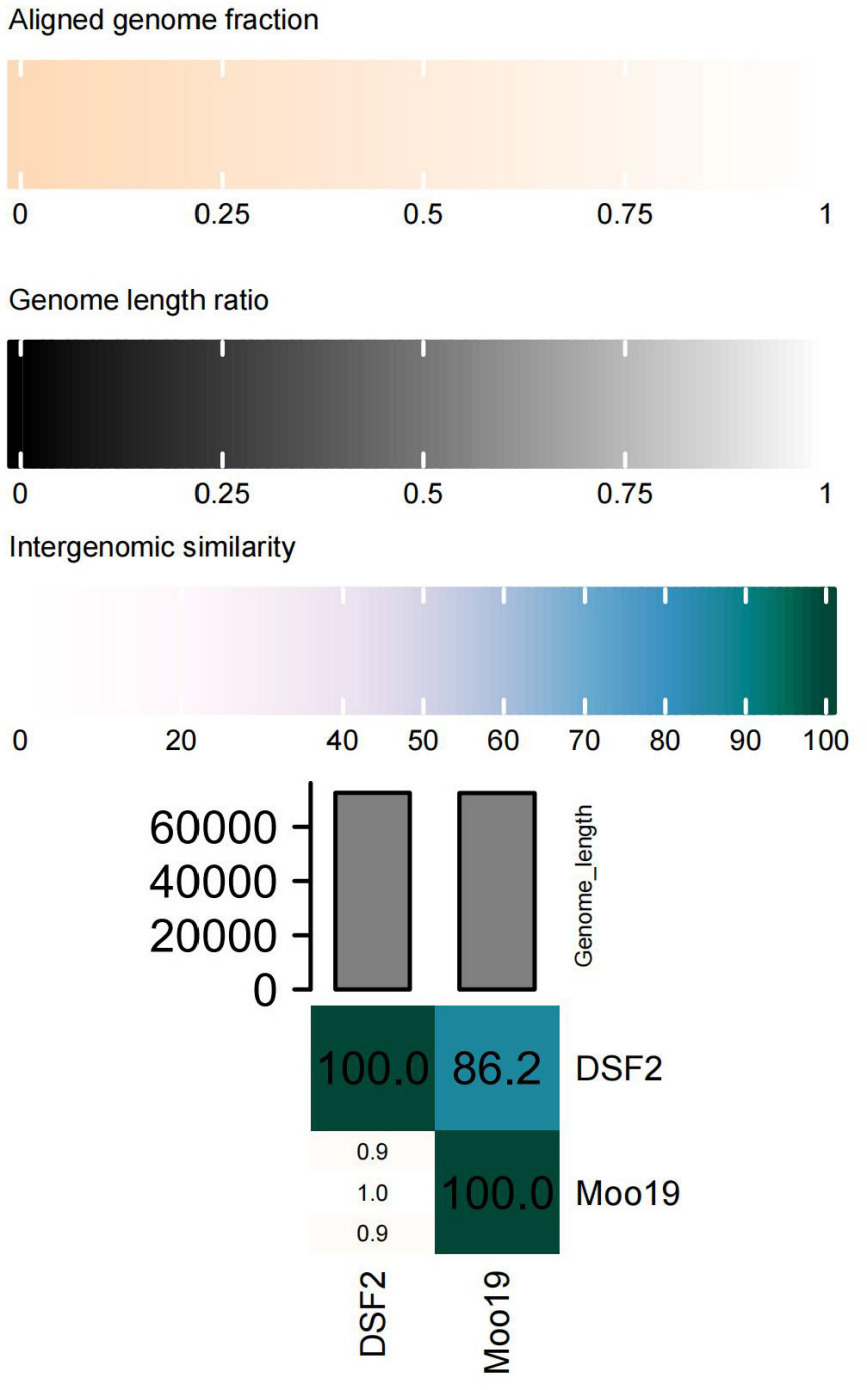
Heatmap of genome comparison between DSF2 and Moo19 based on VIRIDIC analysis. The square matrix displays the aligned genome fraction, genome length ratio, and intergenomic similarity between DSF2 and Moo19.

BLASTn analysis and reference to the latest ICTV guidelines established that DSF2 is classified as a member of the class Caudoviricetes, order Caudovirales, family Schitoviridae, subfamily Enquatrovirinae, and genus Moovirus. DSF2 shares the highest nucleotide sequence similarity with Moo19 (GenBank accession NC_070850.1), with 91.00% coverage and 94.10% identity. Subsequent analysis using the VIRIDIC tool demonstrated that DSF2 and Moo19 exhibited a genome length ratio of 1.0 and an aligned genome fraction of 0.9. The intergenomic similarity within the aligned regions was calculated to be 86.2% (Figure 4). According to the Bacterial and Archaeal Viruses Subcommittee, viruses with less than 5% nucleotide difference are considered the same species (Adriaenssens and Brister, 2017). Thus, DSF2 is designated as a new phage species.

**Figure 5:**
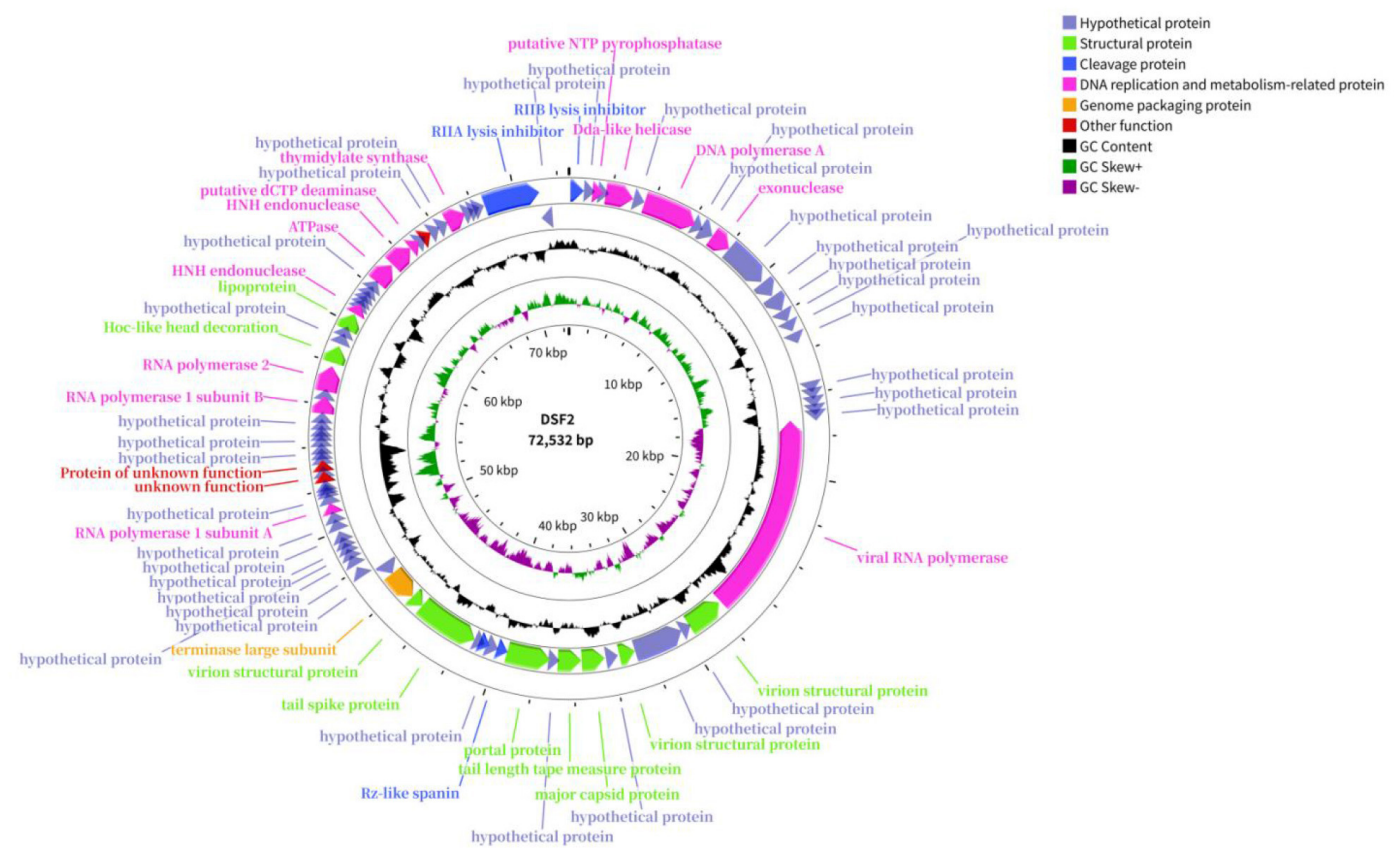
Schematic map of the DSF2 genome generated by Proksee. From inner to outer rings: genome scale, GC skew, GC content (black outward = higher than average; black inward = lower than average), coding sequence (CDS) orientation, and functional categorization of ORFs (colored).

The genome of DSF2 was fully sequenced and was available in GenBank (accession PV699627). Based on predictions by PhageAI and PhaBOX, DSF2 was classified as a lytic phage, with a confidence score of 99.99% in PhageAI and a high-confidence prediction in PhaBOX. Genomic analysis indicated that DSF2 was a double-stranded circular DNA phage with a length of 72,532 bp and a G+C content of 44.89%. A total of 89 open reading frames (ORFs) were predicted, each commencing with an ATG start codon. Among these, 67 were forward-oriented and 18 were reverse-oriented, with forward-oriented ORFs predominating. Four predicted ORFs (ORF18, ORF61, ORF65, and ORF89) lacked annotation in the NR database. Of the remaining 85 ORFs, 54 (63.5%) encoded hypothetical proteins, 2 (2.3%) were of unknown function, and 29 (34.2%) were predicted as functional proteins. These were classified into four functional categories: structural, lytic, DNA replication and metabolism, and genome packaging proteins (Table 2, Figure 5). Neither tRNA nor rRNA genes were detected. No known virulence factors or antibiotic resistance genes were identified in DSF2, suggesting its suitability for further development as a potential therapeutic agent.

**Table 2:** Open reading frame (ORF) analysis of DSF2.

Query_name	Start–End	Size (aa)	Hit_description	Identity (%)	*e*-value
1^+^	64–699	211	RIIB lysis inhibitor [*Shigella* virus Moo19]	99.05	5.44*e*−134
2^+^	759–11,154	131	Hypothetical protein gp50 [*Shigella* virus Moo19]	99.24	5.32*e*−84
3^+^	1,171–11,539	122	Putative NTP pyrophosphatase [*Escherichia* phage AlfredRasser]	69.67	1.45*e*−49
4^+^	1,549–11,680	43	Hypothetical protein F22_0044 [*Escherichia* phage vB_Eco_F22]	92.86	6.47*e*−25
5^+^	1,720–33,030	436	Dda-like helicase [*Shigella* virus Moo19]	99.54	0
6^+^	3,039–33,569	176	Hypothetical protein gp53 [*Shigella* virus Moo19]	100.00	1.49*e*−123
7^+^	3,580–66,159	859	DNA polymerase A [*Shigella* virus Moo19]	98.84	0
8^+^	6,156–66,467	103	Hypothetical protein gp55 [*Shigella* virus Moo19]	98.06	1.59*e*−65
9^+^	6,469–77,125	218	MAG: hypothetical protein [*Siphoviridae* sp.]	83.94	1.03*e*−137
10^+^	7,122–88,102	326	Exonuclease [*Shigella* virus Moo19]	99.39	7.48*e*−244
11^+^	8,099–110,255	718	Hypothetical protein gp59 [*Shigella* virus Moo19]	99.03	0
12^+^	10,311–111,066	251	Hypothetical protein gp60 [*Shigella* virus Moo19]	99.20	8.56*e*−176
13^+^	11,106–111,897	263	Hypothetical protein gp61 [*Shigella* virus Moo19]	98.10	1.42*e*−180
14^+^	11,897–112,457	186	MAG: hypothetical protein [*Siphoviridae* sp.]	96.77	5.98*e*−128
15^+^	12,459–112,956	165	Hypothetical protein gp63 [*Shigella* virus Moo19]	92.12	9.46*e*−62
16^+^	13,200–113,445	81	Hypothetical protein gp64 [*Shigella* virus Moo19]	68.75	5.75*e*−33
17^+^	15,489–115,911	140	Hypothetical protein gp65 [*Shigella* virus Moo19]	98.57	1.88*e*−95
19^+^	16,153–116,650	165	Hypothetical protein E20_59 [*Escherichia* phage E20]	85.19	5.44*e*−105
20^+^	16,652–116,888	78	Hypothetical protein gp66 [*Shigella* virus Moo19]	98.72	1.53*e*−47
21^+^	16,913–117,161	82	Hypothetical protein PP763_gp87 [*Escherichia* phage vB_EcoP_SP5M]	93.83	2.57*e*−50
22^−^	17,196–227,605	3,469	Viral RNA polymerase gp67 [*Shigella* virus Moo19]	97.27	0
23^−^	27,697–229,658	653	Virion structural protein [*Shigella* virus Moo19]	91.12	0
24^−^	29,671–330,078	135	Hypothetical protein gp69 [*Shigella* virus Moo19]	93.33	9.67*e*−81
25^−^	30,140–332,782	880	Hypothetical protein gp70 [*Shigella* virus Moo19]	87.17	0
26^−^	32,788–333,627	279	Virion structural protein [*Shigella* virus Moo19]	97.13	1.93*e*−170
27^−^	33,705–334,343	212	Hypothetical protein gp72 [*Shigella* virus Moo19]	86.32	1.17*e*−108
28^−^	34,404–335,609	401	MAG TPA: major capsid protein [*Podoviridae* sp.]	92.02	1.41*e*−270
29^−^	35,622–336,857	411	Tail length tape measure protein [*Shigella* virus Moo19]	95.86	1.09*e*−240
30^−^	36,876–337,238	120	Hypothetical protein gp75 [*Shigella* virus Moo19]	98.33	1.36*e*−74
31^−^	37,253–339,535	760	Portal protein [*Shigella* virus Moo19]	98.95	0
32^−^	39,544–440,044	166	Rz-like spanin [*Shigella* virus Moo19]	96.39	1.62*e*−104
33^−^	40,025–440,660	211	Hypothetical protein gp78 [*Shigella* virus Moo19]	96.67	1.28*e*−148
34^−^	40,647–440,913	88	Holin [*Shigella* virus Moo19]	97.73	3.11*e*−56
35^−^	40,898–441,227	109	Hypothetical protein gp80 [*Shigella* virus Moo19]	99.08	1.04*e*−66
36^−^	41,338–444,595	1,085	Tail spike protein [*Shigella* virus Moo19]	94.11	0
37^−^	44,586–445,290	234	Virion structural protein [*Shigella* virus Moo19]	98.29	2.12*e*−172
38^−^	45,297–446,889	530	Terminase large subunit [*Shigella* virus Moo19]	99.81	0
39^−^	46,882–447,571	229	Hypothetical protein gp85 [*Shigella* virus Moo19]	99.56	3.47*e*−155
40^+^	47,717–448,034	105	Hypothetical protein gp86 [*Shigella* virus Moo19]	94.29	6.17*e*−64
41^+^	48,281–448,691	136	Hypothetical protein gp1 [*Shigella* virus Moo19]	97.06	1.76*e*−98
42^+^	48,663–449,013	116	Hypothetical protein gp2 [*Shigella* virus Moo19]	81.82	9.36*e*−63
43^+^	49,006–449,290	94	Hypothetical protein gp3 [*Shigella* virus Moo19]	93.62	1.32*e*−62
44^+^	49,290–449,565	91	Hypothetical protein gp4 [*Shigella* virus Moo19]	75.82	3.73*e*−45
45^+^	49,586–449,891	101	Hypothetical protein gp5 [*Shigella* virus Moo19]	92.08	2.52*e*−65
46^+^	50,155–550,475	106	MAG TPA: hypothetical protein [*Podoviridae* sp.]	81.13	3.64*e*−50
47^+^	50,588–550,797	69	Hypothetical protein P4b_00040 [*Klebsiella* phage VLCpiP4b]	75.38	7.12*e*−26
48^+^	50,924–551,307	127	RNA polymerase 1 subunit A [*Shigella* virus Moo19]	97.64	3.80*e*−82
49^+^	51,442–551,726	94	MAG: hypothetical protein [*Siphoviridae* sp.]	66.30	7.66*e*−44
50^+^	51,846–551,965	39	Hypothetical protein gp14 [*Shigella* virus Moo19]	88.46	8.87*e*−07
51^+^	51,962–552,153	63	Hypothetical protein gp15 [*Shigella* virus Moo19]	98.41	7.99*e*−41
52^+^	52,135–552,296	53	Hypothetical protein gp16 [*Shigella* virus Moo19]	79.59	3.27*e*−20
53^+^	52,506–552,739	77	Unknown function [*Shigella* virus Moo19]	98.70	1.23*e*−46
54^+^	52,739–552,963	74	MAG: hypothetical protein [*Siphoviridae* sp.]	93.24	4.38*e*−43
55^+^	52,963–553,319	118	MAG TPA: Protein of unknown function (DUF2493) [*Podoviridae* sp.]	79.66	8.57*e*−68
56^+^	53,322–553,606	94	MAG: hypothetical protein [*Siphoviridae* sp.]	91.49	9.69*e*−56
57^+^	53,603–53,785	60	Hypothetical protein [*Escherichia* phage ST4]	88.33	3.70*e*−29
58^+^	53,782–54,096	104	Hypothetical protein gp20 [*Shigella* virus Moo19]	97.12	2.72*e*−71
59^+^	540,93–54,473	126	Hypothetical protein [*Escherichia* phage ST4]	92.06	4.46*e*−85
60^+^	54,466–54,651	61	Hypothetical protein [*Escherichia* phage N4]	67.21	7.18*e*−23
62^+^	54,874–55,254	126	Hypothetical protein EpBp4_0082 [*Escherichia* phage Bp4]	92.86	3.37*e*−87
63^+^	55,254–55,517	87	Hypothetical protein PP768_gp03 [*Escherichia* phage vB_EcoP-ZQ2]	82.76	1.70*e*−48
64^+^	55,570–56,388	272	RNA polymerase 1 subunit B [*Shigella* virus Moo19]	99.26	5.63*e*−196
66^+^	56,604–57,821	405	RNA polymerase 2 [*Shigella* virus Moo19]	97.53	8.25*e*−297
67^+^	57,985–58,809	274	Hoc-like head decoration [*Shigella* virus Moo19]	94.53	3.64*e*−170
68^+^	59014–59343	109	MAG: hypothetical protein [*Siphoviridae* sp.]	92.16	1.04*e*−65
69^+^	59,447–59,647	66	Membrane protein [*Shigella* virus Moo19]	86.36	4.07*e*−31
70^+^	59,640–60,476	278	Lipoprotein [*Shigella* virus Moo19]	97.84	4.90*e*−168
71^+^	60,600–60,893	97	HNH endonuclease [*Shigella* virus Moo19]	95.88	3.79*e*−65
72^+^	60,890–61,099	69	Hypothetical protein IME11_52 [*Escherichia* phage IME11]	58.82	1.75*e*−22
73^+^	61,096–61,362	88	MAG TPA: hypothetical protein [Phage sp.]	43.02	3.54*e*−16
74^+^	61,395–61,556	53	Hypothetical protein gp34 [*Shigella* virus Moo19]	96.23	6.55*e*−31
75^+^	61,584–61,913	109	Hypothetical protein gp35 [*Shigella* virus Moo19]	80.70	1.22*e*−57
76^+^	61,906–62,265	119	Hypothetical protein gp36 [*Shigella* virus Moo19]	89.08	2.27*e*−77
77^+^	62,277–63,332	351	ATPase [*Shigella* virus Moo19]	98.29	2.57*e*−259
78^+^	63,378–64,508	376	HNH endonuclease [*Shigella* virus Moo19]	99.20	2.91*e*−289
79^+^	64,508–65,014	168	Putative dCTP deaminase [*Escherichia* phage ST4]	94.05	1.10*e*−112
80^+^	65,024–65,221	65	Hypothetical protein gp40 [*Shigella* virus Moo19]	98.46	3.37*e*−34
81^+^	65,298–65,558	86	Immunity to superinfection [*Shigella* virus Moo19]	97.67	8.09*e*−49
82^+^	65,674–66,135	153	Hypothetical protein gp42 [*Shigella* virus Moo19]	98.39	5.32*e*−86
83^+^	66,138–66,584	148	Hypothetical protein gp43 [*Shigella* virus Moo19]	95.95	3.42*e*−95
84^+^	66,581–67,525	314	thymidylate synthase [*Shigella* virus Moo19]	96.18	1.00*e*−226
85^+^	67,586–67,831	81	Hypothetical protein gp45 [*Shigella* virus Moo19]	87.80	1.55*e*−44
86^+^	67,890–68,108	72	Hypothetical protein gp46 [*Shigella* virus Moo19]	94.44	3.81*e*−43
87^+^	68,074–68,385	103	MAG: hypothetical protein [*Siphoviridae* sp.]	70.30	1.64*e*−42
88^+^	68,520–71,144	874	RIIA lysis inhibitor [*Shigella* virus Moo19]	92.91	0

+, forward coding; –, reverse coding.

### 3.6 Comparative genomic analysis

**Figure 6:**
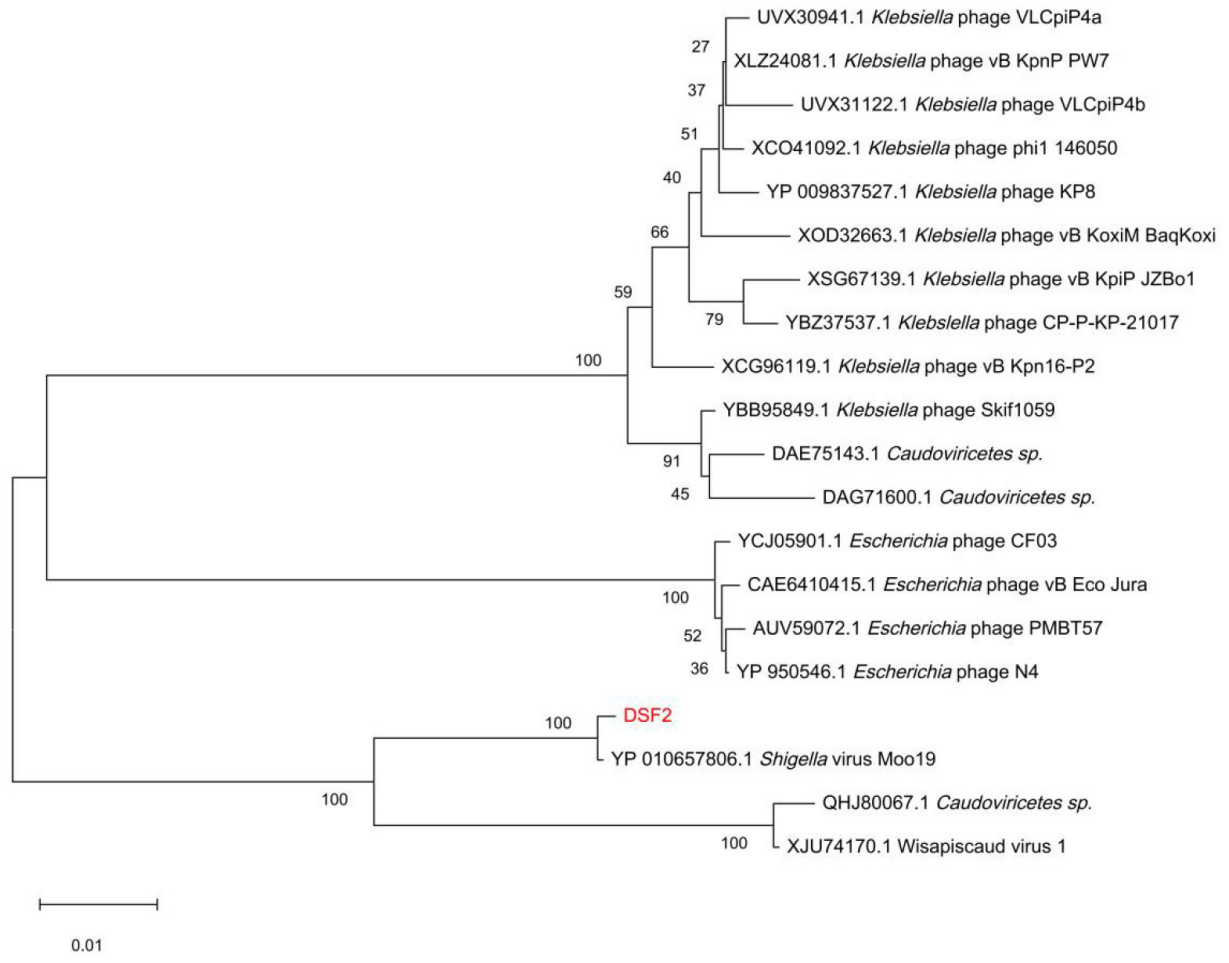
Phylogenetic tree of DSF2 based on the TerL. Scale bar = 0.10 (represents the amino acid substitution rate per site). Bootstrap analysis was conducted with 1,000 replicates and trees were generated using the neighbor-joining method in MEGA 12.0.

Phylogenetic analysis based on the conserved TerL gene (Figure 6) indicated that DSF2 is most closely related to *Enterobacteriaceae* phages, with its nearest relative being *Shigella* virus Moo19.

**Figure 7:**
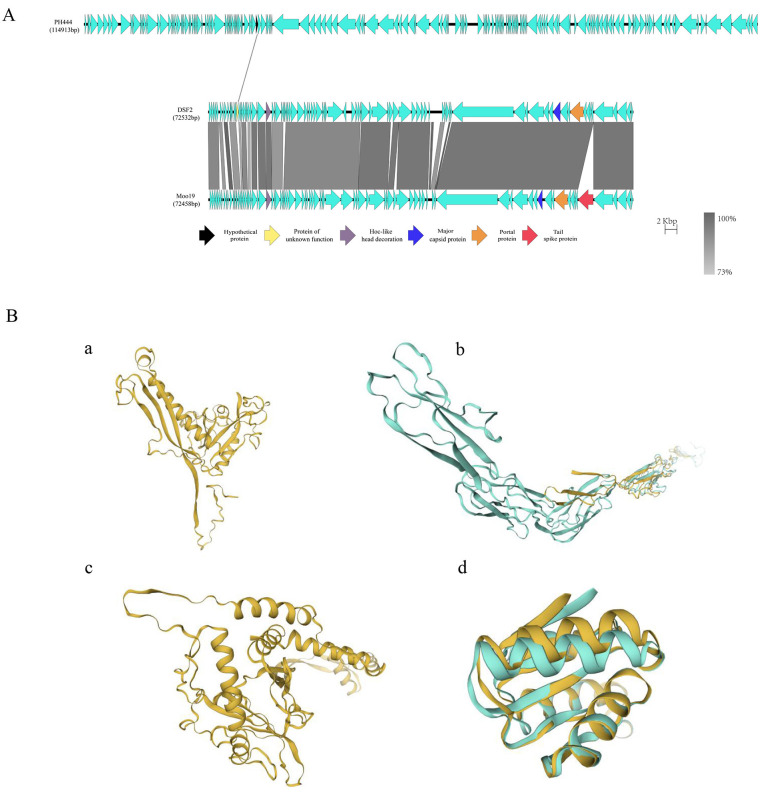
**(A)** Genomic alignment of DSF2, Moo19 and PH444. Arrows represent CDS, with homologous regions highlighted in gray shading. CDS potentially influencing the morphology of the phage head are marked with distinct colors. The color coding is as follows: black for hypothetical protein, yellow for protein of unknown function, purple for Hoc-like head decoration, blue for MCP, red for TSP, and orange for portal protein. The scale bar represents 2 kb, and the percent identity is indicated on the right. **(B)** Three-dimensional structure comparison of proteins potentially influencing phage head morphology. Protein structure comparison is shown for two chains, with each chain highlighted in a distinct color. If the protein structures are highly similar, only one chain's color is shown, and the structures of the chains overlap. **(a)** and **(c)** display the structural comparison of the MCP and portal protein between DSF2 and Moo19. Both structures exhibit high sequence and structural similarity for one chain. **(b)** shows the comparison of the Hoc-like head decoration between DSF2 and Moo19, while **(d)** illustrates comparison of the DSF2 unknown function protein and the PH444 hypothetical protein. In **(b)** and **(d)**, the protein sequence identities are 94.53% and 41.03%, respectively, but the structural differences are significant, with two chains displayed in distinct colors.

Genomic comparisons between DSF2, Moo19, and PH444 revealed that DSF2 and PH444 share only one similar CDS, which encodes an unknown function protein in DSF2 and a hypothetical protein in PH444, with a sequence identity of 41.03%. In contrast, DSF2 and Moo19 exhibit high similarity, with Moo19 possessing a unique CDS encoding the tail spike protein (TSP) (Figure 7A). CDSs potentially affecting phage head morphology, including Hoc-like head decoration, major capsid protein (MCP), and portal protein, show high sequence identity of 94.53%, 99.50%, and 98.95%, respectively. Further comparison of the protein structures indicates significant structural differences between the proteins encoded by the similar CDSs of DSF2 and PH444. Then the Hoc-like head decoration protein structure of DSF2 and Moo19 differs notably, while the MCP and portal protein structures are nearly identical (Figure 7B).

## 4 Discussion

The global rise in MDR *Shigella* imposes a substantial burden on public health due to the resultant increase in diarrheal diseases. Studies have shown that phages, which employ unique mechanisms distinct from conventional antibiotics, offer a potential strategy for combating shigellosis (Ahamed et al., 2023; Choudhary et al., 2024). However, the characterization and genomic analysis of *Shigella*-specific phages remain limited. As of 2020, only 78 *Shigella* phage sequences had been deposited in GenBank (Subramanian et al., 2020) with a mere 17 isolated from China, and just 35 more in the last 5 years. In this work, we isolated and systematically characterized a novel lytic phage, DSF2, capable of infecting MDR *S. flexneri*.

Phylogenetic analysis based on the TerL indicates a close relationship between DSF2, *E. coli*, and *Klebsiella* phages, suggesting a shared evolutionary lineage, although DSF2 is most similar to Moo19. Moo19's isolation from cow pasture water in the United States and high sequence similarity to DSF2 may reflect complex global phage evolution (Chen et al., 2020). Notably, DSF2 constitutes a new species within the *Shigella* phages, as demonstrated by its nucleotide difference (>5%) compared with its closest relative, Moo19. According to ICTV guidelines, DSF2 is classified in the family Schitoviridae and order Caudovirales. Based on predictions by PhageAI and PhaBOX, DSF2 was classified as a lytic phage with no detected antibiotic resistance or virulence factors. These features indicate its promise as a biocontrol candidate for shigellosis management.

DSF2 formed plaques with diameters of 1–2 mm under standardized 12h incubation conditions, consistent with previous studies on *Shigella* phages (Ahamed et al., 2023; Choudhary et al., 2024). The presence of a halo surrounding the plaque formed by DSF2 may be associated with depolymerase activity, which also suggests the potential to disrupt bacterial biofilms (Su et al., 2025). This phenomenon typically arises from the enzymatic degradation of exopolysaccharides in the bacterial capsule or biofilm matrix, allowing phage progeny to diffuse beyond the primary lysis zone and penetrate dense biofilm structures (Visnapuu et al., 2022), thereby highlighting DSF2's therapeutic promise against biofilm-associated shigellosis.

Research indicates that the Siphoviridae, Podoviridae, and Myoviridae morphological classifications remain valid and informative, despite the International Committee on Taxonomy of Viruses discontinuing their use as the basis for taxonomic classification schemes in 2022 (Turner et al., 2023). TEM revealed that DSF2's head shape deviates from the typical icosahedral structure of Podoviridae, such as Moo19, and instead closely resembles the elongated cylindrical form of phage PH444. Further genomic comparisons reveal that DSF2 shares a low sequence identity (41.03%) with PH444 in a single CDS, which encodes proteins of unknown function in DSF2 and a hypothetical protein in PH444. Morphology of the phage head is primarily governed by MCP scaffolds and portal proteins during procapsid assembly, which dictate core lattice geometry via conserved polymerization interfaces, and head decoration proteins like Hoc post-assembly, which decorate the phage head surface to modulate its symmetry and stability (Rao et al., 2023; Kuhn and Thomas, 2022). Hypothetical proteins with such low identity rarely harbor essential structural domains for capsid modulation, typically serving accessory roles in replication, lysis, or metabolism (Van den Bossche et al., 2014). DSF2 exhibits high similarity with Moo19, with their most notable CDS difference being Moo9's unique encoding of a TSP, absent in DSF2. This TSP is crucial for host cell recognition and adsorption. However, TSPs typically attach to the tail baseplate and do not directly affect the morphology or structure of the phage head (Chao et al., 2022). Notably, several CDSs related to phage head morphology, such as the MCP, portal protein, and Hoc-like head decoration protein, show extremely high sequence identities (99.50%, 98.95%, and 94.53% respectively) between DSF2 and Moo19. The near-identical nature of the MCP and portal protein structures between DSF2 and Moo19 suggests that these proteins contribute to a similar structural framework. However, the Hoc-like head decoration protein and its structural differences between DSF2 and Moo19 likely account for the unique morphology observed in DSF2. Recent T4 phage studies demonstrate that head expansion entails ~70% inner volume increase via intercapsomer angle modulation, yielding prolate morphologies (Rao et al., 2023), while Hoc fibers—with C-terminal capsid-binding and variable Ig-like domains—attach off-center to gp23 hexamers, stabilizing expanded lattices particularly at symmetry-broken vertices (Fokine et al., 2023). In DSF2 versus Moo19, the 94.53% identical Hoc-like protein likely imposes divergent angular constraints, driving DSF2's elongated prolate head from a conserved isometric scaffold. Nonetheless, the molecular mechanisms driving phage morphogenesis likely involve complex interactions among portal, decorative, and capsid proteins (Reilly et al., 2020; Kuhn and Thomas, 2022) and remain incompletely understood, as exemplified by phage P1 (Gonzales et al., 2022). These mechanisms thus merit continued in-depth investigation.

In this study, DSF2 was found to infect all serotype 2 variants and X *S. flexneri* strains. Given that *E. coli* and *Shigella* share close genetic similarity, six *E. coli* strains were included in the host spectrum analysis. The results demonstrate that DSF2 does not recognize any of the *E. coli* strains. Several phages have been reported to infect both *E. coli* and *Shigella* (Xu et al., 2021; Kim et al., 2021) and some broad-spectrum phages can lyse multiple bacterial species simultaneously, including *S. dysenteriae, E. coli, Vibrio cholerae, Enterococcus saccharolyticus*, and *Enterococcus faecium* (Kumar et al., 2021). The DSF2 host spectrum is narrow when compared with these broad-spectrum phages. Nevertheless, DSF2 has the potential to avoid impacting non-pathogenic *E. coli* and other natural intestinal flora. A potential limitation of this narrow host spectrum is the risk of serotype conversion in the target *Shigella* strain. *Shigella* strains are known to undergo serotype changes (Sadredinamin et al., 2023), which could impact the effectiveness of DSF2 if the target strain alters its surface antigens. To address this, a phage cocktail that includes DSF2, along with phages targeting different *Shigella* serotypes, may help reduce the risk of treatment failure due to serotype conversion (Fang et al., 2023). Phage cocktail can offer a broader host range and increase the chances of successful infection, meanwhile it has been demonstrated that can reduce resistance development (Wandro et al., 2022; Chen et al., 2024).

The one-step growth curve of DSF2 revealed a 60-min latent period and a burst size of 115 PFU/cell. This extended latent period, longer than the typical 20–25 min for T4 phage and 45–50 min for λ phage in *E. coli* (BioNumbers, 2009), likely reflects prolonged intracellular replication, phage component synthesis, or maturation processes within the *Shigella* host. The large burst size indicates that DSF2 increases rapidly and effectively lyses host bacteria. *S. flexneri* strains. Compared to reported *Shigella* phages, such as vB_SflS-ISF001 (53 ± 4 PFU/cell, 20 min latent period) (Shahin and Bouzari, 2018) and Sfin-2/6 (74–265 PFU/cell, 5–20 min) (Ahamed et al., 2023), DSF2 exhibits a moderately high burst size despite its extended 60-min latent period. This balance suggests optimized intracellular assembly in *Shigella*, yielding robust progeny output ideal for biocontrol against MDR strains, surpassing lower-yield phages in therapeutic potential.

Most bacteriophages characterized to date exhibit maximal stability within a near-neutral pH range (approximately 6–8), whereas their infectivity declines sharply under strongly acidic (below pH 3–4) or strongly alkaline (above roughly pH 10–11) conditions (Jończyk et al., 2011). Consistent with this general pattern, DSF2 displayed high stability under near-neutral conditions, while its viability was markedly reduced at both strongly acidic (pH <3) and strongly alkaline (pH > 11) conditions. The pH range in the human gastrointestinal tract is approximately 1–8 (Evans et al., 1988). Oral bacteriophages are often inactivated by gastric acid, resulting in reduced phage titers and diminished therapeutic efficacy, a limitation that also applies to DSF2. However, Recent advances in phage pharmacology focus on improving phage stability and bioavailability, such as using encapsulation or protective agents to help phages survive the stomach and reach higher concentrations at the infection site (Cheng et al., 2026). Most bacteriophages exhibit optimal stability within a temperature range of approximately 4 °C −42 °C, with maximal activity particularly observed between room temperature and human physiological temperature (roughly 20 °C−37 °C) (Jończyk et al., 2011). The results of this study demonstrate that DSF2 maintains stability across 4 °C−50 °C, indicating that orally administered DSF2 can effectively tolerate gastrointestinal tract temperatures. Oral phage therapy faces several other challenges, including bile salts, mucins, and immune factors that compromise phage stability and infectivity in the gut (Scanlan et al., 2019; Huh et al., 2019). These components influence phage transit and adhesion at intestinal sites of *Shigella* infection, while neutralizing antibodies and complement activation further accelerate phage clearance (Champagne-Jorgensen et al., 2023). Evaluating DSF2's tolerance to these stressors will be essential for enhancing its persistence and efficacy in oral therapy.

While DSF2 demonstrates potent lytic activity against MDR *S. flexneri* serotype 2/X strains *in vitro*, its therapeutic efficacy remains untested in relevant animal models or clinical settings, precluding extrapolation to *in vivo* shigellosis treatment. Additionally, the unique elongated head morphology of DSF2 versus Moo19, despite near-identical MCP (99.50%) and portal (98.95%) sequences, is tentatively attributed to Hoc-like protein divergence (94.53% identity); however, this hypothesis requires validation through cryo-EM structural comparisons or targeted gene knockouts. Future work should prioritize *in vivo* efficacy, gut stability against bile/mucins, and high-resolution morphogenetic analyses to fully realize DSF2's biocontrol potential.

## 5 Conclusion

In summary, DSF2 represents a novel Schitoviridae phage with significant potential for controlling MDR *S. flexneri* serotype 2/X strains, filling a critical gap in the limited pool of *Shigella* phages available in China. Its unique elongated head morphology and the high identity of its MCP with Moo19 emphasize the importance of decoration proteins in phage evolution, challenging traditional morphogenetic paradigms. DSF2's narrow host range, targeting pathogens while preserving commensal *E. coli*, along with its biofilm-disrupting depolymerase activity and high burst size (115 PFU/cell), underscores its suitability as a precise biocontrol agent. Integrating DSF2 into phage therapy cocktails holds promise for mitigating serotype conversion, but further *in vivo* validation and delivery optimization are essential steps for advancing this phage toward practical therapeutic applications for shigellosis.

## Data Availability

The datasets presented in this study can be found in online repositories. The names of the repository/repositories and accession number(s) can be found in the article/supplementary material.
